# Remarks on Du Bouchet’s Nostrum

**Published:** 1849-01

**Authors:** A. Berry

**Affiliations:** Grand Gulf, Miss.


### REMARKS ON DU BOUCHET'S NOSTRUM.

BY A. BERRY, D. D. S.—*Grand Gulf, Miss.*

The last number of the Dental News Letter contains Du Bouchet's offer to the profession and others of "his Odontalgic Drops for the *immediate cure* of toothache." It has two important qualifications to make it pay—the astonishing and the mysterious.

"The effects of the Odontalgic Drops are instantaneous—the most violent cases are cured in a moment." Extraction will *cure* the toothache, and Sulp. Ether, Creosote, or Oil of Cloves may *relieve* it suddenly; but the Drops cure instanter, but will not "destroy the nerve in a tooth become exposed by the extent of caries." It is harmless in its nature, "incapable to produce any pernicious effects upon the living economy," notwithstanding it is death on toothache; even "its taste is agreeable," so that it would not be strange if children cry for it; "and it imparts a pleasant odor to the breath," so that ladies may substitute it for Eau de Cologne.

His modus operandi is not explained, but we are informed that it is "entirely different from that of all similar preparations," and that "it does not cause inflammation of the living membrane, but on the contrary allays such inflammation." It will also remove tenderness in decayed teeth, by repeated application for two or three days, so that they can be "excavated with perfect tolerance."

Well may we regard the discoverer of these Drops as one of the greatest benefactors of our race. We have great admiration for his benevolence, that, after keeping the knowledge of his wonderful remedy for the "hell o' all diseases" within the sanctum of his own breast for a number of years, he has at length placed it within the reach of all interested, for the trifling sum of five dollars. For the death-dealing Letheon and Chloroform we have no longer any use. The fear and trembling with which we were wont to enter the dentist's office are past forever. Forceps and tooth-keys shall frighten us no more. Hereafter, when our teeth ache, we shall apply the "Drops," and then a lasting farewell to the pains and penalties of diseased teeth.

But we would most respectfully say to the author of the sublime art of curing the toothache without destroying the nerve, *Cui bono?* An exposed nerve in a tooth, so long as it possesses vitality, is liable frequently to become painful, from many exciting causes. Toothache should usually be *cured* by extraction, unless it is in the front teeth, on the fangs of which artificial crowns can be sustained by pivots. Any other treatment is commonly but palliative, or beneficial only for a short period. When it is necessary to remove tenderness in teeth, preparatory to filling, we apprehend that extract of nut-gall, applied for a few days, will be found as useful as any agent. When inflammation of the periosteum of a tooth occurs, the only means proper to be employed to allay it, in most cases, is an application of steel. Badly decayed teeth, with their living membranes inflamed, not only produce diseased action in the mouth and other parts of the head, but are prejudicial to the general health.

Light approaches us from the east; but from recent developments it seems that darkness may come from the same point of the compass. We should endeavor to avail ourselves of every important improvement in our profession; but with such discoveries as "Hill's Stoppings," although puffed by reputable practitioners, and the "Odontalgic Drops," puffed by the author, against the permanent utility of which there is prima facie evidence, we should not suffer ourselves to be humbugged.

